# Self-Perceived Benefits of Cognitive Training in Healthy Older Adults

**DOI:** 10.3389/fnagi.2018.00112

**Published:** 2018-04-25

**Authors:** Vina M. Goghari, Linette Lawlor-Savage

**Affiliations:** ^1^Department of Psychology, University of Toronto, Toronto, ON, Canada; ^2^Department of Psychology, University of Calgary, Calgary, AB, Canada

**Keywords:** cognitive training, seniors, expectation, working memory, placebo, cognitive errors

## Abstract

The idea that individualized, computer-based cognitive training improves cognitive functioning in non-trained domains is highly contested. An understudied area is whether cognitive training improves one’s own perception of cognitive and day-to-day functioning. Furthermore, no studies have compared working memory training to programs that train higher-level processes themselves, namely logic and planning, in improving perception of cognitive abilities. We investigated self-reported changes in: (a) cognitive errors relevant to daily life; (b) expectations regarding training; and (c) impact of training on daily life, in healthy older adults who completed working memory training or logic and planning training. Ninety-seven healthy older adults completed 8-weeks of computerized cognitive training that targeted either working memory or logic and planning. Findings were compared to a no-training control group. Participants reported fewer cognitive failures relevant to daily life after training compared to the no-training control group, with a greater reduction in errors reported by the logic and planning training group compared to the working memory training group. Trainees’ perception of training efficacy decreased over time. Nonetheless, approximately half of the participants in both training groups endorsed “some improvement” or more in self-perceived day-to-day functioning at post-testing. These results support the conclusion that individualized computerized cognitive training may enhance subjective perceptions of change and that higher level cognitive training may confer additional benefits. Findings suggest that cognitive training can enhance cognitive self-efficacy in healthy seniors.

## Introduction

Maintaining cognitive functioning is a noteworthy concern of aging adults, and is important in promoting independence, good mood and quality of life (Langlois et al., 2013; Boss et al., 2015; Halil et al., 2015). Subjective perceptions of aging can influence seniors’ health and survival (Westerhof et al., 2014). As such, adults concerned about their cognitive proficiency often look to online cognitive training programs to enhance or maintain their cognitive abilities (Fernandez, 2011). However, whether or not cognitive training enhances untrained cognitive functioning in healthy adults is a contentious area of investigation (Melby-Lervåg et al., 2016). Furthermore, the role of psychological factors (e.g., belief in improvement after participating in an intervention aimed to improve cognitive functioning) are understudied in the cognitive training literature, and are just as critical as understanding the more “objective” cognitive benefits of training. The present study investigated the impact of two domain-specific computerized cognitive training protocols, working memory and logic and planning, compared to a no-training passive control group, on self-reported outcomes in healthy older adults.

The general goal of cognitive training is to practice computerized tasks and have that activity transfer to improvements in daily life; however, a fundamental question, which has long plagued cognitive training literature is, simply, does cognitive training benefit cognition? Studies which utilize objective measures of performance consistently show improved performance on target games, and often, improvements on tasks within the same cognitive domain as that which was trained, an effect termed near-transfer (Melby-Lervåg et al., 2016). However, improvements on tasks within cognitive domains not targeted by training, termed far transfer, is both more impactful and more contested than near transfer. Given the lack of consensus in the literature about whether working memory training generalizes to gains in far transfer, and the debate about the specific methodology by which these claims ought to be tested, the working memory training literature has been deemed “reliably ambiguous” (Melby-Lervåg et al., 2016; Urbánek and Marček, 2016).

When investigating whether or not cognitive training works, the vast majority of studies have focused on measuring objective cognitive changes following training using neuropsychological tests. A smaller body of research has investigated individuals’ own perceptions of cognitive change after different types of cognitive training, with a number of studies pointing to improved self-reported cognition in healthy older adults (Preiss et al., 2010; Stepankova et al., 2012). One notable study of 487 community dwelling adults over the age of 65 compared cognitive training to a general cognitive stimulation program and found that both training groups self-reported increased cognitive functioning irrespective of group membership (Smith et al., 2009). A minority of studies have also investigated whether cognitive training improves day-to-day functioning in older adults, with some studies suggesting no subjective improvement (Ball et al., 2002; McDougall et al., 2010) and others reporting sustained self-reported improvements in instrumental activities of daily living (Willis et al., 2006; Rebok et al., 2014).

Given these inconsistent findings, recent studies have investigated other subjective factors such as participants’ expectations of cognitive training, or more simply, the placebo effect of an intervention. Expectations about the impact of cognitive training may influence effort and persistence on post-training cognitive tests; for example, those who trained may believe they improved, thus, to confirm that belief, they are more effortful on post-testing (Shipstead et al., 2012; Boot et al., 2013). By controlling for differential expectations and including two active training conditions, placebo effects can be more definitively parsed apart from true treatment effects (Boot et al., 2013).

We focused on two forms of cognitive training, working memory training and logic and planning training. Working memory training was chosen because it is a well-studied, domain-specific form of cognitive training. Logic and planning training was chosen as it trains higher level executive skills that are similar to the far transfer cognitive abilities most frequently studied (e.g., non-verbal reasoning and problem solving). Also, we wanted to evaluate whether training higher level abilities themselves, such as logic and planning, would lead to greater perceived cognitive benefit, given that logic and planning abilities may have greater perceived relevance to day-to-day functioning (Bell-McGinty et al., 2002). Furthermore, these two training protocols were chosen because a recent meta-analysis reported promising effects of both working memory training and executive functioning training on cognitive functioning in healthy adults aged 60 years and older (Karbach and Verhaeghen, 2014); albeit, the findings have been contested (Melby-Lervåg and Hulme, 2016).

In a published article on the same sample presented here, we assessed whether training on working memory and logic and planning games improved both near and far transfer cognitive abilities based on objective cognitive tasks (Goghari and Lawlor-Savage, 2017). We found evidence for improvement on the training tasks, but no effects for the near or far transfer on the cognitive tasks. We also found that both groups were equally motivated to participate in training.

Given that self-perceptions regarding one’s own cognitive ability can influence factors such as mood and physical health (Westerhof et al., 2014) we sought to identify, in the same sample as Goghari and Lawlor-Savage (2017), whether working memory or logic and planning training altered self-perception of cognitive ability even in the absence of objective cognitive changes. In the above noted study, participants were not provided with feedback regarding their performance; therefore, any self-reported changes after training should be based solely on their belief of improvement (or lack thereof) after training. Clearly, a perceived improvement in ability (i.e., increased confidence) could have powerful impacts on a positive aging experience.

In the current article, we present the results of self-reported changes in cognitive functioning, as well as expectations regarding training, in healthy older adults who completed working memory training or logic and planning training. We hypothesize that the two training groups, relative to the untrained control group, will report that they expect their cognitive abilities to improve after training, and that they assess their day-to-day cognitive functioning as improved after training. Last, we explore whether the working memory training group, compared to the logic and planning training group, will report greater gains on measures of day-to-day cognitive functioning. The theoretical rationale for testing this exploratory aim is that logic and planning activities, relative to working memory activities, are likely to be perceived as more closely related to day-to-day tasks.

## Materials and Methods

### Participants

Participant characteristics are described in detail in Goghari and Lawlor-Savage (2017). Briefly, healthy community-based adults aged 65 and over were recruited in Calgary, Alberta. Potential participants completed an online screening questionnaire. Exclusion criteria were based on self-report and included: (a) age lower than 65 years; (b) history of inflammation, infection, or injury to the brain; (c) past or present history of neurological or psychiatric illness, dementia, or altered consciousness; (d) recent (within 3-months) benzodiazepine or illicit drug use; (e) current cardiovascular or respiratory problems (e.g., stroke, transient ischemic attack, heart attack, severe asthma, chronic obstructive pulmonary disease); (f) current visual, auditory, or motor impairments; and (g) a Mini Mental State Examination (MMSE) score of less than 27/30. We also required: (h) participants to be proficient in English; (i) be comfortable using a computer; and (j) have access to a computer with high-speed internet connection.

Eligible individuals were invited to participate, and those interested were sent an online consent form and questionnaire assessing demographics and factors which influence cognitive performance (mood, physical activity and sleep quality). Participants completed an in-person cognitive testing session, then were quasi-randomized by sex to one of three groups: working memory training, logic and planning training, or no-training control.

Training group participants were provided with an in-person orientation to the BrainGymmer website and training games. Trainees were instructed to train for approximately 30 min per day, 5 days a week, for 8-weeks, at a time and location of their convenience. Training adherence was monitored and participants were contacted via phone or email if they strayed from the training schedule. After the 8-week training period, all participants completed a second in-person cognitive testing session. Training group participants were entered into one of several draws held throughout the study, but were not otherwise remunerated. The no-training control group participants were paid $25 per testing session. Procedures contributing to this work comply with the ethical standards of the relevant national and institutional committees on human experimentation and with the Helsinki Declaration of 1975, as revised in 2008. The University of Calgary Conjoint Faculties Research Ethics Board approved this protocol and written informed consent was obtained.

### Training Programs

As described in detail previously (Goghari and Lawlor-Savage, 2017), all training games were provided by BrainGymmer^1^. Each training program contained three adaptive games.

#### Working Memory Training

The working memory games targeted maintenance and manipulation of information. The Multi-Memory game required participants to remember the placement of tiles on a grid despite the tiles disappearing and distractor tiles being presented. For each trial, participants recreated the original placement of tiles. Grid size and number of tiles changed as a function of performance. The Moving Memory game required participants to choose matching pairs of cards after they were shown, then flipped and scrambled. The number of pairs to be remembered changed as a function of performance. Last, in the *N*-back game, a card was shown then hidden. Further cards then appeared, and participants indicated whether the current card was the same as the hidden card presented “n” back. The number of cards to be remembered changed as a function of performance.

#### Logic and Planning Training

These games targeted higher level planning, reasoning and problem solving abilities. In the Square Logic game, participants stacked numbered squares following the rule that the stacked squares were within one digit of each other. The number of squares to stack changed as a function of performance. In Out of Order, a variety of squares were presented showing different shapes, patterns within the shape, color and number of shapes. Trainees had to rearrange squares so that each square matched at least one characteristic of adjacent squares. The number of squares to arrange changed as a function of performance. Last, in Patterned Logic, participants had to choose from a number of pieces to correctly complete a pattern. Pattern complexity changed as a function of performance.

### Demographic and Cognitive Measures

At baseline, participants completed an online self-report demographics questionnaire and inventories of state characteristics that influence cognitive performance (e.g., mood, physical activity levels and sleep quality). Task specifics, including justification for each task chosen, were described in detail in a previously published study (Goghari and Lawlor-Savage, 2017). Briefly, mood was measured with the Beck Depression Inventory-II (BDI-II; Beck et al., 1996) and the Beck Anxiety Inventory (BAI; Beck and Steer, 1990). Physical activity was measured with the Rapid Assessment of Physical Activity (RAPA), which includes aerobic, and strength and flexibility indices (Topolski et al., 2006). Sleep was measured with the Pittsburgh Sleep Quality Index (PSQI; Buysse et al., 1989). We measured mood, physical activity, and sleep to document scores between groups, as these factors are also associated with cognition. In-person, objective cognitive testing consisted of psychometrically valid tests of general cognitive ability, working memory, processing speed, reasoning, and executive functioning. The MMSE (Folstein et al., 1975) was used as a measure of mental status, and the Wechsler Abbreviated Scale of Intelligence-II (WASI-II; Wechsler, 2011) was administered as an estimate of general cognitive ability. The effect of training on all objective cognitive tasks is presented in a previous study (Goghari and Lawlor-Savage, 2017).

### Self-Report Training-Related Measures

To assess subjective perception of cognitive changes, all participants completed the Cognitive Failures Questionnaire (CFQ) at baseline and post-training (Broadbent et al., 1982). The CFQ is a 25-item self-report questionnaire in which participants indicate how often (options range from “*never*” to “*very often*”) they experience specific cognitive errors or frustrations in day-to-day life. The questionnaire focuses on errors or slips in perception (e.g., “Do you fail to hear people speaking to you when you are doing something else?”), memory (e.g., “Do you forget why you went from one part of the house to another?”), and motor functioning (e.g., “Do you drop things?”). The CFQ is correlated with both self- and collateral-ratings of cognitive mistakes and is well correlated with other self-report measures of memory and attention difficulties (Broadbent et al., 1982). Higher scores indicate more cognitive errors.

Participants in training groups also completed the Motivation and Expectations questionnaire (created for this study) before and after training to compare pre-training expectations to post-training self-perceptions. Four questions specifically assessed participants’ expectations and perceptions before and after training. First, participants indicated the extent to which they expected to improve from training on a scale ranging from 0 = *no expectation/impact* through to 7 = *substantial expectation/impact*. Prior to training, participants answered the question “In terms of performance on the cognitive training tasks, how much do you think you might improve?” After training, the question was: “In terms of your performance on the cognitive training tasks, how much do you think you improved overall?”

Second, participants were asked to report the extent (0 = none, 7 = substantial) to which they believed the training program they were assigned to *targeted* memory, language, planning/organization, speed of processing, attention and visual/spatial ability. The third question asked to what level (0 = none, 7 = substantial) they believed their training program was expected to *improve* (baseline question) or succeeded in *improving* (post-training question) their memory, language, planning/organization, speed of processing, attention and visual/spatial ability.

The fourth question on the Motivation and Expectation questionnaire assessed expectations and perceptions regarding real world outcomes. At baseline, participants were asked “Do you think this cognitive training will impact your daily life (e.g., ability to remember phone numbers, remember meetings and tasks)?” and after training, “Do you think this cognitive training impacted your daily life (e.g., ability to remember phone numbers, remember meetings and tasks)?” One further question regarding self-reported motivation was previously described and reported, with no significant differences between the training groups (Goghari and Lawlor-Savage, 2017).

### Statistical Analysis

Demographic, mood, physical activity, sleep, baseline cognitive and cognitive training data were analyzed with correlations and chi-squared analyses. Baseline data were compared between groups using analysis of variance (ANOVA) and chi-square tests. To analyze pre- to post-training changes between the two training groups, we used Bayesian Repeated Measures Analyses of Variance (Bayes RM-ANOVAs). To analyze self-reported changes in the CFQ among all three groups before and after training, a 3 (group: working memory training, logic and planning training, no-training control) × 2 (time: pre, post) Bayes RM-ANOVA was conducted. Support for the alternative hypothesis was followed-up with 2 (group) × 2 (time) Bayes RM-ANOVAs with means and standard deviations reported when needed to illustrate group differences.

Bayesian analyses were conducted using the JASP statistics package, version 0.7, available online at https://jasp-stats.org/ (Love et al., 2015). JASP utilizes default prior probabilities in calculating Bayes factors, which provides a probability estimate for support of the null hypothesis (_01_). Specific procedures of the Bayesian RM-ANOVA approach, including the use of default priors, have been described elsewhere (e.g., Masson, 2011; Rouder et al., 2012; Jarosz and Wiley, 2014). We followed the interpretation process described by Sprenger et al. (2013). To summarize, *BF*_01_ > 5 denotes evidence that the null hypothesis is true, a *BF*_01_ 3–5 provides support for the null hypothesis and a *BF*_01_ > 1.25 to <3 suggests weak support for the null hypothesis. Regarding the alternative hypothesis, a *BF*_01_ < 0.05 provides evidence that the alternative hypothesis is true, a *BF*_01_ 0.33–0.05 suggests that the alternative hypothesis is supported, and a *BF*_01_ < 0.8 to <0.33 indicates weak support for the alternative hypothesis. A *BF*_01_ ranging from 0.08 to 1.25 is considered inconclusive and that either hypothesis could be true.

## Results

### Participant Characteristics

**Figure 1:**
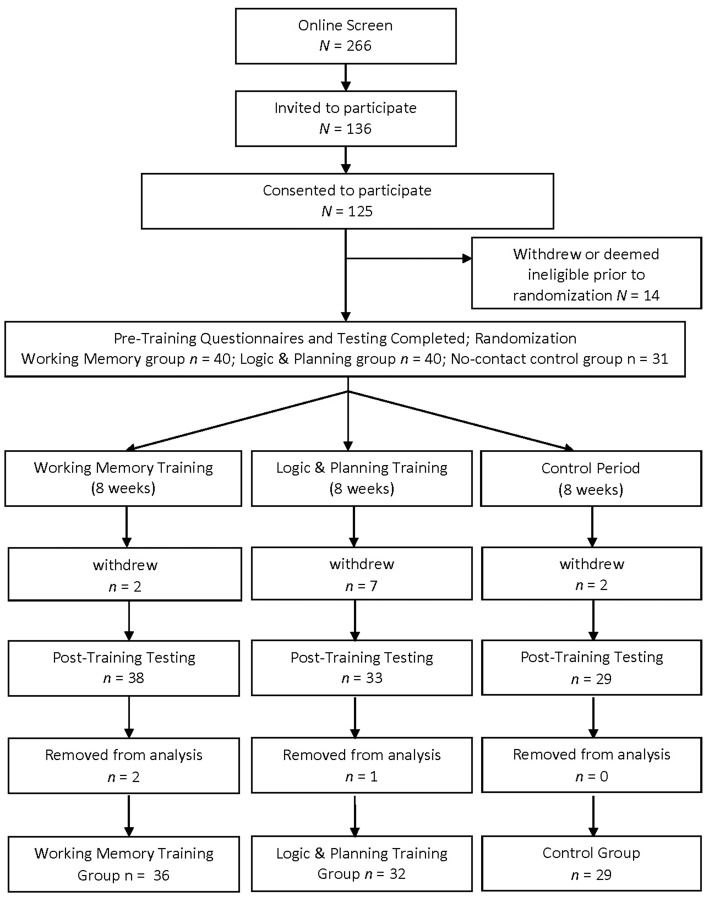
Flow chart of study design. Reproduced from Goghari and Lawlor-Savage (2017).

Participant characteristics and training data are presented in Table 1 and are the same as those presented in our previous article on this sample (Goghari and Lawlor-Savage, 2017). Briefly, 125 participants initially consented, 14 withdrew or were deemed ineligible prior to randomization, 11 withdrew during the study, and data for three participants were removed due to poor training compliance (see Figure 1). Common reasons for withdrawal or low compliance were disliking the training or experiencing a health difficulty. Final analyses included 36 working memory trainees, 32 logic and planning trainees and 29 no-training control participants.

**Table 1:** Participant Characteristics.

	Working memory training	Logic and planning training	Passive no training control
**Demographics**			
*N*	36	32	29
Age	70.39 (4.54)	70.81 (4.98)	70.24 (4.48)
Range	65–86	65–84	65–78
Sex (% female)	64	69	69
Ethnicity (% Caucasian: Asian: Other)	94: 6: 0	88: 13: 0	86: 7: 7
Marital status (% coupled)	72	69	62
Education (years completed)	15.43 (3.48)	15.44 (2.86)	15.52 (2.86)
Range	7–23	10–21	9–22
Employment (% retired)	86	81	83
Income (% <$50,000: $50,000–95,000: >$95,000)	31: 47: 22	55: 23: 23	32: 50: 18
**Mood, Sleep, Physical Activity**			
Beck Depression Inventory	5.61 (6.55)	3.66 (4.29)	4.97 (6.12)
Range	0–24	0–18	0–26
Beck Anxiety Inventory	3.33 (4.42)	2.25 (3.22)	2.48 (3.00)
Range	0–18	0–15	0–10
PSQI Total	4.86 (3.03)	4.47 (3.07)	4.41 (3.09)
Range	2–12	0–12	0–15
RAPA Aerobics	4.83 (1.75)	5.56 (1.27)	5.14 (1.60)
Range	0–7	4–7	2–7
RAPA Strength and Flexibility	1.56 (1.30)	1.69 (1.26)	1.62 (1.24)
Range	0–3	0–3	0–3
**Cognition**			
MMSE	28.89 (0.95)	28.67 (1.00)	29.03 (0.94)
Range	27–30	27–30	27–30
WASI-II 4-Item Composite	111.75 (13.24)	113.94 (10.28)	112.52 (11.88)
Range	66–133	91–135	96–148
**Cognitive Training**			
Training Time (h)	19.01 (2.14)	19.44 (2.42)	
Range	14.23–22.68	12.32–24.87	

*Note. PSQI, Pittsburgh Sleep Quality Index; RAPA, Rapid Assessment of Physical Activity; MMSE, Mini Mental State Examination; WASI-II, Wechsler Abbreviated Scale of Intelligence-II*.

Participants were similar in age (*F*_(92,94)_ = 0.13, *p* = 0.88), sex distribution ($X_{\left(2\right)}^{2}$ = 0.25, *p* = 0.88) and education level (*F*_(2,94)_ = 0.08, *p* = 0.99). Of note, groups were also similar for self-reported depression (*F*_(2,94)_ = 1.00, *p* = 0.37), anxiety (*F*_(2,94)_ = 0.83, *p* = 0.44), physical activity subscores (*F*’s = 0.09–1.86, *p*’s = 0.16–0.91) and sleep quality (*F*_(2,94)_ = 0.21, *p* = 0.81). Groups had similar MMSE scores (*F*_(2,92)_ = 1.10, *p* = 0.34) and WASI full scale IQ scores (*F*_(2,94)_ = 0.29, *p* = 0.75).

### Training Characteristics

For the two training groups, cognitive testing occurred a mean of 1.25 days (SD = 1.72) before training start and 4.18 days (SD = 5.31) after training concluded. The two groups did not differ in terms of number of days between baseline testing and training (*F*_(1,66)_ = 0.32, *p* = 0.58) or number of days between the end of training and completing post-training tests (*F*_(1,66)_ = 1.23, *p* = 0.26). Training groups trained for a similar number of hours (*F*_(1,66)_ = 0.62, *p* = 0.43). Important, participants within each training group attained a higher level of difficulty after training on every game played (*p* < 0.001).

### Target of Cognitive Training Protocols

As part of the Motivation and Expectations questionnaire, participants in both training groups were asked to endorse which cognitive skills they thought the training was targeting. Working memory trainees (100%) were more likely than logic and planning trainees (62.5%) to indicate that training was meant to increase memory abilities (χ^2^ = 16.39, *p* < 0.001). Conversely, the logic and training group (71.9%) was more likely than the working memory training group (30.6%) to endorse planning/organization as a training target (χ^2^ = 11.60, *p* = 0.001). Neither group greatly endorsed language as a perceived target (working memory group 8.3%, logic and planning group 9.4%; χ^2^ = 1.33, *p* = 0.25). Both groups believed processing speed (working memory group 86.1%, logic and planning group 93.8%; χ^2^ = 1.07, *p* = 0.30), attention (working memory group 69.4%, logic and planning group 75.0%; χ^2^ = 0.26, *p* = 0.61) and visual/spatial abilities (working memory group 77.8%, logic and planning group 84.4%; χ^2^ = 0.48, *p* = 0.49) were targets of training.

### Self-Reported Expectations Regarding Cognitive Training

Means and standard deviations of the self-reported expectation data based on the Motivation and Expectations questionnaire are presented in Table 2. The extent to which participants in each group, at baseline, reported expecting to improve on the training tasks was compared with Bayesian ANOVAs. The hypothesis, that at baseline the groups had similar expectations for improvement on training tasks, was supported, albeit weakly (*BF*_01_ = 2.10).

**Table 2:** Means and Standard Deviations before (T1) and after (T2) training period, and Bayes factors^¶^ of time and interaction effects.

Item	WMT T1 mean (*SD*)	WMT T2 mean (*SD*)	LPT T1 mean (*SD*)	LPT T2 mean (*SD*)	PC T1 mean (*SD*)	PC T2 mean (*SD*)	Group (*BF*_01_)	Time (*BF*_01_)	Group × Time (*BF*_01_)
Improvement on trained	4.44 (1.44)	3.89 (0.98)	4.84 (1.19)	4.41 (0.95)	–	–	0.87	0.03	0.11
tasks									
Impact on domains:									
Memory	–	3.53 (1.32)	–	3.50 (1.59)	–	–	4.00	–	–
Language	–	2.03 (1.28)	–	2.59 (1.72)	–	–	1.45	–	–
Planning	–	2.57 (1.70)	–	3.22 (1.58)	–	–	1.34	–	–
Speed	–	3.44 (1.28)	–	3.78 (1.45)	–	–	2.58	–	–
Attention	–	3.61 (1.42)	–	3.50 (1.63)	–	–	3.86	–	–
Visuospatial	–	3.47 (1.56)	–	4.09 (1.47)	–	–	1.20	–	–
Daily impact	4.49 (1.34)	3.06 (1.54)	4.53 (1.30)	3.22 (1.54)	–	–	4.02	<0.0001	<0.0001
CFQ	37.14 (14.14)	31.67 (10.69)	35.34 (18.41)	26.56 (11.10)	35.31 (14.23)	31.41 (11.34)	4.46	<0.0001	0.006

*Note. WMT, working memory training; LPT, logic and planning training; PC, passive no-training control; CFQ, Cognitive Failures Questionnaire. ^¶^Bayes Factors assessing the null hypothesis (BF_01_) < 0.33 indicates support for the alternative hypothesis (i.e., 3:1 probability in favor of the alternative), and BF_01_ < 0.05 indicates strong evidence for the alternative hypothesis (i.e., 20:1 probability in favor of the alternative). Conversely, BF_01_ > 3 indicates support for the null hypothesis (3:1 probability in favor of the null) and BF_01_ > 5 indicates strong evidence for the null (20:1 probability in favor of the null). Values between 0.33 and 3 suggests weak support for the alternative, and values between 1.25 and <3 suggest weak support for the null. Values close to 1 are not informative, and suggests the two hypotheses are equally likely to be true*.

Bayesian RM-ANOVA was then conducted to identify group differences in self-reported expectations over the training period (i.e., group by time). For expected level of improvement on the training tasks, data supported the alternative hypothesis suggesting an overall effect of time (*BF*_01_ = 0.03), with *lower* scores after training. Data supported the alternative hypothesis regarding a group by time interaction (*BF*_01_ = 0.11). Further examination of this effect indicated that those in the working memory training group reported a larger reduction (mean reduction = 0.55, *SD* = 0.46) over time in level of expected improvement relative to those in the logic and planning group (mean reduction = 0.43, *SD* = 0.24).

Further investigation of frequencies demonstrated that despite lowering of scores over time, especially in the working memory group, a substantial proportion of the participants endorsed *“some improvement”* or more (rating of 4 or greater on a 7 point Likert scale) after training: 75% of working memory trainees (89% at baseline), and 94% of logic and planning trainees (91% at baseline).

### Self-Reported Improvement of Specific Cognitive Domains After Cognitive Training

Investigation of frequency scores of self-reported improvement after training for individual cognitive domains demonstrated that trainees endorsed “*some improvement*” or more to a substantial extent for both groups. For the working memory training, 64% of trainees rated “*some improvement*” or more for memory, 67% for attention, 61% for processing speed and visual-spatial abilities, 34% for planning and organization and 14% for language. For the logic and planning training, 50% of trainees rated “*some improvement*” or more for planning and organization, 69% visual-spatial abilities, 63% for processing speed, 56% for attention and memory and 34% for language.

Next, self-perceived improvement after training was examined for specific cognitive domains between training groups. Support for the null was present, indicating groups did not differ in the extent to which they believed their memory (*BF*_01_ = 4.00) and attention (*BF*_01_ = 3.86) abilities changed after training. Similarly, support for the null was present, albeit weakly, regarding self-perceived improvement after training for language abilities (*BF*_01_ = 1.45), planning/organization (*BF*_01_ = 1.34), speed of processing (*BF*_01_ = 2.58) and visual/spatial ability (*BF*_01_ = 1.20). Collectively, findings suggest that group membership (i.e., training program) was not a significant factor in influencing participants’ beliefs regarding their improvement in specific cognitive abilities after training.

### Self-Reported Impact of Cognitive Training on Daily Life

No baseline differences emerged between groups in self-reported expected impact of training on day-to-day functioning (*BF*_01_ = 3.95). For perceived impact of training on daily life, evidence for the alternative was revealed for a main effect of time (*BF*_01_ < 0.0001) with *lower* scores after training, suggesting that trainees did not improve as much as they expected they would, based on self-report. The group by time interaction indicated evidence for the alternative hypothesis (*BF*_01_ < 0.0001). Further examination of this effect indicated that those in the working memory training group reported a greater decrease from anticipated to perceived impact on daily life (mean decrease = 1.43, SD = 0.20) relative to the logic and planning group (mean decrease = 1.31, SD = 0.24). In other words, logic and planning trainees’ expectations were closer matched with self-perceived outcomes.

However, despite lower scores over time, especially in the working memory group, investigation of frequencies indicated that a substantial proportion of the participants reported that training resulted in at least *“some impact”* on their cognitive abilities: 45% of working memory trainees (89% at baseline), and 50% of logic and planning trainees (84% at baseline).

### Self-Reported Cognitive Failures After Cognitive Training

The CFQ assessed day-to-day self-reported errors in memory, perception and motor functioning with higher scores indicating more errors (Broadbent et al., 1982). Bayesian ANOVA revealed that baseline CFQ scores did not differ among the three groups (*BF*_01_ = 6.33). Bayesian RM-ANOVA was conducted comparing the three groups before and after testing on CFQ scores. For the main effect of time, the data provided evidence for the alternative hypothesis (*BF*_01_ < 0.0001) with lower scores (i.e., fewer self-perceived errors) after training. The data also indicated evidence for the alternative hypothesis of a group by time interaction (*BF*_01_ = 0.006). Three follow-up Bayesian RM-ANOVAs were conducted to specify the effects. First, the working memory group was compared to the no-training control group. Evidence was present for a main effect of time (*BF*_01_ = 0.05), with lower scores after training. Results also indicated weak support for a group by time interaction (*BF*_01_ = 0.51) with the working memory training group showing greater reductions in CFQ scores (mean reduction = 5.47, SD = 3.45) relative to the no-training control group (mean reduction = 3.9, SD = 2.89). Second, the logic and planning group was compared to the no-training control group. Data provided evidence for a main effect of time (*BF*_01_ = 0.01) with lower scores after training. There was weak support for an interaction (*BF*_01_ = 0.52), with the logic and planning group reporting lower CFQ scores (mean reduction = 8.78, SD = 7.31) relative to the no-training control group. Finally, the working memory and the logic and planning groups were compared to each other. Evidence was present for a main effect of time (*BF*_01_ = 0.001), with lower scores after training, and for a group by time interaction (*BF*_01_ = 0.005). The logic and planning group had greater reductions in CFQ scores than the working memory group. Collectively, findings indicate that trainees reported experiencing fewer cognitive errors in their daily life after participating in cognitive training compared to untrained participants, with logic and planning trainees reporting greater benefits from training relative to the working memory trainees.

## Discussion

The cognitive training literature has a limited, albeit increasing, focus on the effects of subjective appraisals and psychological factors on cognitive training-related outcomes. To this end, we compared two active training conditions, working memory and logic and planning training, on self-reported expectations and self-reported cognitive errors relevant to day-to-day functioning, before and after training. Inclusion of two active conditions and a no-training control group, at least on the CFQ, allowed us to control for demand characteristics that could be associated with self-report measures. Additionally, we chose to train higher level abilities themselves, namely logic and planning, to investigate whether that would have additional benefits to self-perceived outcomes.

We found that participants who completed cognitive training reported fewer cognitive failures relevant to day-to-day life (based on CFQ responses) compared to untrained controls. This finding is consistent with other studies in the field that find better self-reported cognition in healthy older adults who complete a cognitive training intervention (Smith et al., 2009; Preiss et al., 2010; Stepankova et al., 2012). This study furthers this literature by comparing two more domain-specific cognitive training protocols. Of note, our findings also showed that the logic and training group reported fewer cognitive failures than the working memory training group. Given that we measured multiple factors that can be associated with cognition (e.g., mood, physical activity and sleep), and revealed no differences in these factors between groups, it is more likely that aspects of the particular training programs account for differential effect. These findings could be due to a number of reasons. First, the logic and planning training targeted higher level cognitive abilities such as reasoning and problem-solving, tasks which are strongly linked to the cognitive failures that are experienced in daily life by healthy seniors (Bell-McGinty et al., 2002). Second, the logic and planning training group rated themselves as having greater improvement on the training tasks than the working memory training group, which could have created a greater expectation of change in other areas of functioning (Zahodne et al., 2014). Third, for generalizability to how cognitive training is accessed in the real world, we used an online cognitive training platform. The purpose of the different cognitive training games was to measure separate cognitive processes; however, that means other factors, such as differential difficulty and complexity, between the two types of cognitive training could influence our results. Nevertheless, these findings suggest that training higher-level skills themselves could potentially provide an additional benefit to self-perceptions of cognitive functioning. Last, the no-training control group also reported few cognitive failures from the pre- to post-assessment. This could be a sample specific finding or, alternatively, it could be that be participating in pre-post cognitive training studies creates a response-style of reduced scores on follow-up. Regardless, this finding underscores the importance of including a passive no-training control group in cognitive training studies focusing on self-report of cognition.

Consistent with the cognitive failures data, we found that a substantial number of participants (50% or greater) in both groups rated themselves as having at least *“some improvement”* or more in attention, visual/spatial skills, processing speed and working memory after training. Furthermore, 50% of participants in the logic and planning group also reported at least “some improvement” or more in their logic and planning skills after training. The abilities that participants reported more substantial self-perceived changes align with the domains the participants thought were being trained.

Unexpectedly, in this study we found that participants’ experience did not match their original expectations. Trainees rated their perceived improvement on the training tasks as lower after training, compared to their pre-training anticipated improvement. This drop in ratings from expected to perceived improvement was greater for the working memory training group compared to the logic and planning training group. Similarly, participants’ expectations of the impact of training on their day-to-day life became lower over the course of training in both groups, with a greater decrease in the working memory training group relative to the logic and planning training group. The decrease in ratings was quite substantial, from 89% at baseline to 45% at follow-up for the working memory trainees, and 84% at baseline to 50% at follow-up for the logic and planning trainees. In other words, participants were expecting to improve more from training. These findings suggest that participants may have questioned the relevance of practicing these cognitive tasks. This is surprising as the games were adaptive, a feature meant to continually push participants’ cognitive ceiling, in line with theories of adult cognitive plasticity as related to cognitive training (Lövdén et al., 2010). Also, the working memory training participants reported lower expectations for improvement on the training tasks after training than the logic and planning training groups. This could be due to the more specific nature of the working memory training tasks compared to the broader logic and planning training tasks, (e.g., difficulty and complexity) or the engagement of the specific tasks utilized. However, this finding does appear to suggest that higher level training could additionally benefit expectations of improvement. Regardless, approximately half of the trainees reported some improvement in day-to-day functioning, a noteworthy finding. Given that expectations can influence participant effort and persistence on post-training cognitive tests leading to enhanced performance and greater scores at post-testing, increasing expectations of change could be a powerful psychological process worth harnessing in cognitive training protocols (Shipstead et al., 2012; Boot et al., 2013; Foroughi et al., 2016).

As reported previously (Goghari and Lawlor-Savage, 2017), this sample of participants improved over the training period on the specific tasks trained. However, as noted above, participants rated their perceived improvement on the task as lower than they expected, which is in contrast to objective measures showing that they did in fact improve. This finding is noteworthy as it highlights that objective and subjective improvements are not always congruent, in line with recent meta-analytic findings (Crumley et al., 2014). Although not explicitly measured, it could be that participants lacked self-efficacy in terms of the actual cognitive task, which influenced their subjective ratings. Furthermore, although neither group significantly improved on objective cognitive measures, training participants in this study still reported improvements in their real-world cognitive functioning, based on the CFQ, a reliable and valid measure of perceived cognitive functioning in daily life (Broadbent et al., 1982). It is possible that the CFQ was a more accurate and clearer measure of perceived benefits of cognitive training than was the Motivation and Expectations questionnaire created for this study. Overall, these findings underscore the necessity to investigate subjective cognitive change in addition to objective cognitive change.

The importance of increasing perceived cognitive ability in older adults has been thoroughly demonstrated. First, others have demonstrated that in older adults, confidence in their cognitive abilities is associated with improvements in cognitive performance, mediated by greater task persistence (Beaudoin and Desrichard, 2017). Conversely, negative beliefs regarding aging (e.g., social stereotypes, self-perceived decreases in functioning) are associated with poorer cognitive functioning, and more broadly, decreased physical health and longevity (Levy et al., 2012, 2014; Westerhof et al., 2014). More alarmingly, recent results suggest that negative beliefs regarding aging are associated with increased biomarkers of neurodegeneration (i.e., hippocampal volume loss, neurofibrillary tangle and amyloid plaque accumulation; Levy et al., 2016). Thus, if cognitive training is able to improve subjective perception of cognition in healthy seniors, irrespective of changes in objective cognition, such an effect may be beneficial regarding long-term health-related outcomes. That being said, we acknowledge that there are likely more direct approaches to facilitate the improvement of cognitive confidence, such as psychoeducational interventions and strategy-based training programs.

In summary, this study provides some evidence for improved self-perceived cognitive performance and improved self-reported cognitive functioning relevant to daily life for healthy adults who participated in cognitive training compared to those who did not train. Furthermore, this study provides support that training higher level abilities such as logic and planning may have further benefit for subjective cognition and expectations of change. Given the conflict and controversy regarding whether cognitive training leads to objective cognitive change in healthy older adults, future research should focus on participants’ self-perceived benefits. Self-perceived benefits of cognitive training are an important outcome that, as an adjunct to other useful measures, may be a potential mechanism for improving daily life and overall longevity in healthy seniors.

## Author Contributions

VMG developed the protocol, trained and supervised the research assistants, supervised data collection and wrote large parts of the manuscript. LL-S conducted the analyses and wrote parts of the manuscript with input from VMG.

## Conflict of Interest Statement

The authors declare that the research was conducted in the absence of any commercial or financial relationships that could be construed as a potential conflict of interest.

## Footnotes

^1^https://www.braingymmer.com/en/
